# Boracic Acid in the Treatment of Leucorrhœa

**Published:** 1888-02

**Authors:** 


### *BORACIC ACID IN THE TREATMENT OF LEUCORRHEA.*

For months past, I have made frequent use of boracic acid in the treatment of leucorrhea in a manner hitherto unmentioned, at least so far as has come under my notice, and with surprising success; in every case where I applied it, prompt and permanent improvement resulted.

Having had some excellent results from the boracic acid packing in chronic suppurative otitis, I determined to resort to its use in a similar way in a case of leucorrhea, which had for several months resisted a most persevering use of the regular orthodox remedies—*i. e.*, nitrate of silver, tincture of iodine, fluid hydrastis and bismuth, hot water irrigations, etc. The experiment was eminently successful, and the patient returned home within a fortnight well and happy, and has so remained ever since—many months—during which time I have had occasion to resort to the remedy frequently, and with uniformly good results.

My manner of using it is as follows: Having first irrigated the vagina at as high a temperature as can well be borne by the patient, a cylindrical speculum is introduced, and the vaginal wall very carefully dried—first with a soft sponge, and then with absorbent cotton. This done, boracic acid in crystals is poured into the mouth of the speculum, and pushed up against the uterus and vault of the vagina with a clean cork caught in a uterine sponge-carrier, sufficient acid being used to surround and bury the intravaginal portion of cervix, filling the upper part of vagina. A tampon of absorbent cotton is then firmly pressed against the packing, and held *in situ* until the folds of the vaginal walls close over it as the speculum is withdrawn.

This should be allowed to remain three or four days, or even longer, as after this time there still remain some undissolved particles of the acid; nor will the tampon seem at all offensive. The ostiom vaginæ, if examined in twenty-four hours, instead of being besmeared with the leucorrheal secretion or discharge, presents a clean appearance, and bathed in a watery fluid which begins to appear several hours after the packing has been placed; and, in my cases, this was the only discharge noticed afterward.

However, a second, or even a third, repetition may be necessary; but in none of my cases, numbering nearly a score, have I found more than a second packing called for, and in many one sufficed; and in no instance has it occasioned pain, not even inconvenience. I do not claim for this agent and method infallibility, nor should constitutional dyscrasias be ignored, and this local treatment be depended on, unaided, to effect a cure; but here, as in the treatment of leucorrhea by other remedies, a proper association of all means having a curative influence upon the disease, constitutes the rational therapeutics. My individual experience with this remedy in the treatment of leucorrhea, though limited to few cases, to establish its universal efficacy, if such a wide range of power can be claimed for any medicine at any time, none the less proves it as one of the agents which, when properly employed, promises much in the treatment of the annoying and, sometimes, intractable conditions constituting the pathology of leucorrhea, particularly when the change is in the vaginal glands or mucous membrane, or from intracervical inflammation. Nor will harm likely result from its use, though it fail in maintaining the place my experience would give it.—*Schwartz, in St. Louis Cour. of Med.*

